# Empathy and the Public Perception of Stillbirth and Memory Sharing: An Australian Case

**DOI:** 10.3389/fpsyg.2018.01629

**Published:** 2018-08-31

**Authors:** Christina J. Keeble, Natasha M. Loi, Einar B. Thorsteinsson

**Affiliations:** School of Psychology, University of New England, Armidale, NSW, Australia

**Keywords:** stillbirth, empathy, memory sharing, public perception, empathy-attitudes-action model

## Abstract

**Objective:** Stillbirth devastates families and leaves them struggling to grieve the death of their baby in a society that expects grief symptoms to decrease over time. Previous research has suggested that increased memory sharing opportunities can lead to positive mental health outcomes. The aim of the current study was to examine people’s perceptions of stillbirth as well as the perceived appropriateness of affected parents sharing memories of their child. In addition, we examined whether manipulating empathy would have an effect on people’s perceptions of stillbirth.

**Method:** Participants included 200 Australian men and women 18 to 74 years of age (*M* = 36.76, *SD* = 12.59) randomly allocated to one of three experimental conditions (i.e., low empathy, high empathy, and control). The high empathy group watched a video about stillbirth and was instructed to imagine how the people portrayed felt; the low empathy group watched the same video but was instructed to remain detached; and the control group watched an unrelated video. Participants were then asked how much money they would be willing to donate to a fictional stillbirth organization, followed by the completion of questionnaires measuring (a) perceptions of stillbirth, (b) empathy, and (c) the appropriateness of parents sharing memories of a stillborn child with different groups of people over time.

**Results:** The empathy manipulation had an effect on empathy and the willingness to help effected parents (high empathy vs. control). However, empathy did not have an effect on participants’ perceptions toward stillbirth nor appropriateness of sharing memories. The appropriateness of sharing memories decreased as time passed and social distance increased.

**Discussion:** Individuals who have experienced stillbirth need to be aware that societal expectations and their own expectations in relation to sharing memories may not correspond to each other and that they may need to educate their social group about their need to share memories. Removing the taboo surrounding stillbirth is vital for both parents and those to whom they would wish to communicate.

## Introduction

The public perception in high income countries is that stillbirth does not happen often (Flenady et al., 2011), however, Australian statistics tell a different story. Stillbirth, as defined in Australia, is the death of a baby of at least 20 weeks gestation or 400 g birth weight (Hilder et al., 2014). In 2012, there were 2,255 reported stillbirths in Australia resulting in 7.2 fetal deaths per 1,000 births (Hilder et al., 2014). Despite these high numbers, it remains a highly under-researched area (Frøen et al., 2011). Most expecting parents are not aware that stillbirth is even an option as a birth outcome, that lifestyle factors can increase the incidence of stillbirth, or that many stillbirths are potentially preventable (Flenady et al., 2011).

The effect of stillbirth on families and society comes at a high cost. In high income countries like Australia, one in five mothers, following a stillbirth, suffers from significant long-term depression, anxiety, or post-traumatic stress disorder (PTSD; Frøen et al., 2011). Christiansen et al. (2013) found that persistent PTSD symptoms continued long after the loss in both parents (up to 18 years). Following a stillbirth, higher levels of depression and anxiety in subsequent pregnancies are found in more recently bereaved mothers and those who felt a lack of social support when their baby was stillborn (Hughes et al., 1999; Turton et al., 2001). The effect of stillbirth on subsequent children puts them at risk of less desirable interactions with their mother (Turton et al., 2009) and disorganized attachments in subsequent children have been found to stem from unresolved grief issues in the mother (Turton et al., 2004). The effects of stillbirth impacts the entire family unit and can stress the couple relationship (Brierley-Jones et al., 2014).

Social stigma and loss of identity is commonly experienced by mothers of stillborns (e.g., Murphy, 2012; Cacciatore et al., 2013; Brierley-Jones et al., 2014; Wonch Hill et al., 2017). The struggle of whether to disclose the stillbirth or not is echoed in the literature by mothers who have experienced guilt, shame, social isolation, and exclusion from family, friends, colleagues, and strangers (Cacciatore, 2010; Thompson, 2013; Brierley-Jones et al., 2014). A survey of Australian parents affected by stillbirth found that they believed others felt the baby was a taboo subject, the mother should try to forget and have another baby, and the stillbirth was “natural selection” and therefore never meant to live (Frøen et al., 2011). Brierley-Jones et al. (2014) argued that the social stigma experienced by mothers occurred even for those who had worked through their grief; they were still subject to negative reactions regarding their experience, thus making it a social phenomenon.

The families of stillborns are faced both with their grief and the task of finding a social identity for their baby. Peelen (2009) explained the process of developing a social identity for a new baby is slow and generally occurs after birth, leaving stillborn babies omitted. Images of stillborn babies are one way a social identity for the baby can be facilitated and their place in the family sphere acknowledged (Godel, 2007).

The making of memories for families of stillborns begins in the hospital with the rituals parents choose to practice following the birth (Layne, 2000; Kobler et al., 2007; Crawley et al., 2013). Items like footprints and photographs, and rituals such as holding and talking about the baby or posting to memorial sites on social media (e.g., Facebook; Hayman et al., 2018), serve to facilitate the grief process, create memories and an identity for the baby as well as validating their experience as parents (Bleyen, 2010; Basile and Thorsteinsson, 2015). No research in Australia appears to have investigated people’s perceptions of the appropriateness of such practices by parents of stillborns or the extent to which they are shared with others.

Previous research has shown the benefits of memory making for families of stillborns (Cacciatore et al., 2008; Ryninks et al., 2014; Basile and Thorsteinsson, 2015). Other studies have indicated adverse effects, including increased PTSD symptoms connected to memory making, resulting from contact with the stillborn child (Hughes et al., 2002; Hughes and Riches, 2003). Research conducted by Crawley et al. (2013) was the first of its kind to investigate the association of sharing memories of the stillborn and its effect on maternal mental health. They found that PTSD and anxiety was lower in those with greater memory sharing and higher in those who desired to speak more about their baby, but felt they could not (Crawley et al., 2013). Similar research in the grief literature indicates that continued opportunities to share are important (Diamond et al., 2012).

The grief literature indicates that people expect there to be an end point to the period of grief, and that grief symptoms should decrease over time (Costa et al., 2007; Penman et al., 2014). These findings lead to the question of what people think is appropriate to share and for how long. If the opportunities to share memories of their stillborn babies increases maternal wellbeing, if there is a social or perceived stigma surrounding stillbirth, and if there is a possibility of an expected finite grieving period, overcoming those barriers are necessary to facilitate sharing opportunities.

Inducing empathy for a person in a stigmatized group can lead to improved attitudes toward the entire group (Batson et al., 2002). Empathy, as defined by Batson (1991), is an other-oriented, vicarious emotion produced by taking the perspective of a person perceived to be in need. Batson et al.’s (2002) research has led to an established, empathy-attitudes-action model whereby empathy for an individual in a stigmatized group leads to helping action for the entire group.

Replicating past results (Batson et al., 1997), Batson et al. (2002) utilized the empathy-attitudes-action model to increase helping behavior for a highly stigmatized group, heroin addicts. While parents of stillborns are not considered a highly stigmatized group in the same way as heroin addicts, they are potentially subject to social or perceived stigma. Thus the benefit of inducing members of the public to empathize with their experience could potentially lead to more positive social interactions for the parents which could facilitate the grieving process.

The results of Frøen et al.’s (2011) study indicates that increased understanding and support for parents is associated with more accurate societal knowledge of the causes of stillbirth. Raising awareness can lead to increased funding and research toward stillbirth with the overall goal being to reduce the incidence of stillbirth. According to Frøen et al. (2011), stillbirth prevention strategies will be most effective if aligned with strategies to provide better support and understanding for women.

The following hypotheses are proposed.

Compared to those in a low empathy or control condition, the induction of empathy (i.e., high empathy condition) would result in: (Hypothesis 1) more positive perceptions toward those who have experienced stillbirth; (Hypothesis 2) more group helping behavior as measured by the allocation of more funds to help; and (Hypothesis 3) responses showing higher levels of appropriateness of sharing for between one and five years post-loss. Hypothesis 4 predicted that the appropriateness of sharing with another person would get progressively less appropriate as the relationship distance between the protagonist and the other person grew. Finally, hypothesis 5 predicted that the perceived appropriateness of sharing memories would decrease with the passage of time for those in the low empathy or control conditions compared to those in the high empathy condition.

## Materials and Methods

### Participants

An Australian sample of 510 participants undertook this online experimental study. Three participants completed the survey but did not give consent to use their data and were thus excluded. After excluding those with incomplete survey data, 200 adults aged 18 years and over were retained (39.2% retention rate). The participants comprised 29 males and 171 females aged 18 to 74 years (*M* = 36.76, *SD* = 12.59), for other demographics see **Table 1**. Participants were randomly allocated to one of three conditions: high empathy (*n* = 69; 34.5%), low empathy (*n* = 75; 37.5%), or control (*n* = 56; 28%).

**Table 1:** Demographics (*N* = 200).

Variable	*n*	%
Sex		
Male	29	14.5
Female	171	85.5
Education		
Didn’t finish high school	4	2.0
Completed year 10	14	7.0
Completed year 12	34	17.0
TAFE/diploma	63	31.5
Bachelor’s degree	46	23.0
Postgraduate diploma	19	9.5
Master’s degree	16	8.0
Doctoral degree	4	2.0
Parental Status		
Never had children	76	38.0
Have living children	97	48.5
Have deceased children	5	2.5
Have living and deceased children	22	11.0
Personal experience of loss		
Miscarriage	65	32.5
Stillbirth	6	3.0
Child loss	10	5.0
None of the above	124	62.0
Family or friend’s experience of loss		
Miscarriage	140	70.0
Stillbirth	80	40.0
Child loss	75	37.5
None of the above	33	16.5
Marital status		
Single and never married	56	28.0
Married, *de facto*, and living with partner	122	61.0
Divorced, separated, and widowed	21	10.5
Didn’t answer	1	0.5

### Materials

Participants were asked a range of questions relating to their age, sex, marital status, level of education, parental status, and experiences of stillbirth, miscarriage, and child loss.

#### The Perinatal Grief Scale – Short Form (PGS)

The PGS (Potvin et al., 1989) is a 33-item instrument designed to measure perceptions of those who have experienced stillbirth and measured on a 5-point Likert type scale from 1 (*strongly agree*) to 5 (*strongly disagree*). The PGS consists of three subscales including active grief, difficulty coping, and despair. The current study measured others’ perceptions of stillbirth (Plagge and Antick, 2009). Thus the language was modified from first person to third person narrative (e.g., “I feel empty inside” to “She feels empty inside”). The PGS has demonstrated sound construct, convergent, and external validity (Toedter et al., 2001). For the current sample, higher scores reflect more perceived grief (α = 0.91), difficulty coping (α = 0.85), and despair (α = 0.91). The Cronbach’s alpha for the total scale was 0.96.

#### The Emotional Response Questionnaire (ERQ)

The ERQ (Toi and Batson, 1982) consists of adjectives describing different emotional states used to measure empathy. It includes six empathy adjectives: sympathetic, soft-hearted, moved, compassionate, tender, and warm. For each adjective, participants were asked how much they experienced the emotion while watching the video, ranging from 1 (*not at all*) to 7 (*extremely*). The scores were averaged to form an index of empathy (Batson, 1991). For the present study, the Cronbach’s alpha was 0.94.

#### The Memory Sharing Questionnaire (MSQ)

The MSQ asked participants their perceptions of the appropriateness of parents of stillborn babies sharing memories. The types of questions, employed from previous research (e.g., Bremborg and Rådestad, 2013), included talking about the baby, sharing photographs of the baby, and sharing keepsakes of the baby with the following groups of people: each other, family, friends, colleagues, and others. The questions were asked about the appropriateness of sharing during the first year of loss and again at five years post-loss (e.g., “Please choose how often you think it is appropriate, within the first year of loss, for the parents of stillborn babies to talk about the baby with friends?” and “Please choose how often you think it is appropriate, five years after the loss, for the parents of stillborn babies to talk about the baby with friends?”). The questions were measured on a 5-point Likert scale from 1 (*never*) to 5 (*very often*). Subscales were calculated by totaling all of the “each other” (or “family”, “friends, “colleagues”, or “others”) responses for the talk, sharing photographs, and sharing keepsakes questions. The Cronbach’s alpha for *within the first year of loss* was 0.94 and for *five years post-loss*, 0.97. The subscales’ alphas ranged from 0.88 to 0.95.

### Procedure

Participants were recruited for the study via a survey link posted on social media (e.g., Facebook) and a variety of internet forums (e.g., forums.whirlpool.net.au) in order to reach the general Australian population. The project was approved by the Human Ethics Committee of the University of New England (Approval No HE15-091).

Once implied consent was obtained for each participant, they were randomly allocated to one of three conditions: high empathy, low empathy, or control. This study required the use of deception to obtain desired results and was based on Batson’s framework (Batson et al., 2002).

Secondly, participants in the high empathy group watched a 5 min, 30 s video entitled, “Stillbirth Stories” (Raising Children Network, 2014, November 3) which showed a couple discussing their experience of stillbirth. The participants were given instructions to imagine how the parents would feel and to feel the full impact of what they had been through (Batson et al., 2002). Those in the low empathy group watched the same video as the high empathy group but were given instructions to remain objective and detached while watching the video. Finally, participants in the control group watched a 5 min, 20 s video entitled, “How to Make a Quilt using a Sudoku Pattern” (Smith, 2014, October 23) and were given no viewing instructions. The experimental groups’ video was chosen as the information was presented in a fairly neutral, matter-of-fact manner on a highly emotive topic. The control group’s video was chosen as it was unlikely to induce any empathy.

Thirdly, participants were provided with a fictional scenario explaining that they would be participating in a community survey by the IST Fund (a non-government organization that funds not-for-profit organizations) to get the public’s input into the allocation of how their funds should be distributed. They were told that the annual funds for all groups is capped at AU$150,000 in a financial year and that the maximum amount a single organization can receive is AU$50,000. The previous financial year had seen three groups receive funding. The survey was to determine if a fourth fictional organization, the Australian Stillbirth Association (ASA), would be accepted and how much money should go to it. Allocating money to the fictional group meant that the three existing groups (early intervention for children with special needs, single parent education, and animal rights) who wished to continue to receive funds at the same level would lose or have their funding reduced. The participants were asked to decide if this new organization, the ASA, should be included and how much money should be allocated from AU$0 to AU$50,000 in increments of AU$5,000. They received information on the ASA as an organization that supports families in making memories up to five years post-loss along with raising awareness of stillbirth. After making a decision regarding the allocation of funds, participants completed the PGS, ERQ (which acted as a manipulation check for the experience of empathy), and MSQ.

Finally, participants were given a debriefing sheet explaining why deception was used and the option to go into a draw to win an AU$100 gift voucher. If participants decided to participate, they were taken to a different survey that was not linked to the research survey thus keeping the responses anonymous.

## Results

### Measuring Perceptions of Stillbirth and Empathy (Hypothesis 1)

A one-way between groups ANOVA for the effect of group (i.e., control, low, and high empathy) on perceptions of stillbirth (as measured by the PGS) revealed no significant differences between the groups when empathy was induced, *F*(2,197) = 0.36, *p* = 0.697, and η^2^ = 0.004.

A one-way ANOVA for the effect of group (i.e., control, low, and high empathy) on empathy (as measured by the ERQ) showed a group (i.e., experimental manipulation) main effect, *F*(2,197) = 35.39, *p* = 0.001, and η^2^ = 0.26. *Post hoc* analyses with Tukey’s HSD revealed that the high empathy condition (*M* = 5.35, *SD* = 1.11) and the low empathy condition (*M* = 5.12, *SD* = 1.34) scored significantly higher on empathy compared to the control condition (*M* = 3.25, *SD* = 2.03; both *ps* < 0.001), indicating that the manipulation of empathy was partially successful. However, there was no significant difference between the high empathy and the low empathy condition, *p* = 0.641, and η^2^ = 0.004.

### Empathy and Group Helping (Hypothesis 2)

A one-way ANOVA, groups (i.e., control, low, and high empathy), was conducted to explore the impact on helping (money allocated in the helping options), *F*(2,197) = 4.97, *p* = 0.008, and η^2^ = 0.05. *Post hoc* analyses with Tukey’s HSD revealed that those in the high empathy group (*M* = AU$38,985 and *SD* = AU$14,465) allocated significantly more money to help than those in the control group (*M* = AU$29,821 and *SD* = AU$16,867; *p* = 0.006). However, there was no significant difference between the allocations of money to help the group between those in the high empathy condition and those in the low empathy condition (*M* = AU$33,533 and *SD* = AU$17,721; *p* = 0.117), nor between those in the low empathy condition and those in the control condition (*p* = 0.408). Empathy scores had a low correlation, in the expected direction, with whether participants believed that the hypothetical organization should receive funding, *r* = -0.14, and *p* = 0.047.

### Memory Sharing With Who? (Hypotheses 3 and 4)

To examine the differences between who it is appropriate to share memories with, we conducted a within-subjects repeated-measures MANOVA with *group* (i.e., control, low, and high empathy) by *sharing with* (i.e., each other, family, friends, colleagues, or others as measured by the MSQ) within both time periods (up to 1 and 5 years). The main effect for group was not statistically significant, Wilks’ lambda = 0.99, *F*(4,392) = 0.46, *p* = 0.768, and η^2^ = 0.005 and neither was the *group* by *sharing with*, Wilks’ lambda = 0.95, *F*(16,380) = 0.58, *p* = 0.899, and η^2^ = 0.024. However, there was a statistically significant effect for *sharing with*, Wilks’ lambda = 0.22, *F*(8,190) = 82.18, *p* < 0.001, and η^2^ = 0.776. All multiple pairwise comparisons (Sidak adjustment) within each year (e.g., family vs. friends in the up to 1 year and friends vs. colleagues at 5 years) showing statistically significant differences, *p* < 0.001. The appropriateness of sharing with another person got progressively less appropriate as the relationship distance between the protagonist and the other person grew (moving from top to bottom in column 1 in **Table 2**).

**Table 2:** Mean (SD) for sharing with different groups of people, 1 and 5 years after stillbirth, repeated-measures ANOVA comparison (*N* = 200).

Sharing	1 year	5 years	*F*	*P*	Hedges’ *g*
Each other	13.30 (2.27)	11.42 (2.78)	147.90	<0.001	0.74
Family	12.53 (2.43)	10.74 (2.96)	137.54	<0.001	0.66
Friends	11.51 (2.65)	9.78 (3.11)	150.51	<0.001	0.60
Colleagues	8.29 (2.86)	7.03 (2.99)	95.27	<0.001	0.43
Others	7.34 (3.01)	6.56 (2.97)	43.05	<0.001	0.26

### Time and Memory Sharing (Hypothesis 5)

A within-subjects repeated-measures MANOVA was run with two levels of *time* (up to 1 and 5 years) for five outcome measures *sharing with* (i.e., each other, family, friends, colleagues, or others). The overall effect was statistically significant, Wilks’ lambda = 0.50, *F*(5,195) = 38.55, *p* < 0.001, and η^2^ = .497, with individual repeated-measures ANOVAs supporting Hypothesis 5 and showing that appropriateness of sharing decreased with time (**Table 2**).

## Discussion

The current study sought to investigate empathy and its effect on the Australian public’s perceptions of stillbirth and willingness to help parents of stillborns. It also sought to compare the effects of empathy and the passage of time on the Australian public’s perceived level of appropriateness of sharing memories about the stillborn baby by the parents.

The viewing instructions had a large effect on the empathy felt by participants in both the high and low empathy groups as compared to the control group, demonstrating that the induction of empathy was effective. While Batson et al.’s (2002) original study did not employ a control group, it was necessary for this study in order to accurately measure if the induction of empathy was having an effect. While no statistically significant difference was found between the high and low empathy groups, this could be due to the manipulation. Principally, simply telling people to be objective may not have been enough. Even though the experimental video was chosen as its format was considered neutral in its presentation, the topic of stillbirth is still highly emotive.

As hypothesized, the induction of empathy had a significant effect on the allocation of money to help the hypothetical organization, with a difference found between the high empathy condition and the control condition. However, no statistically significant differences were found between the high empathy and low empathy conditions or between the low empathy and control conditions. This could potentially be because helping parents who have experienced stillbirth is a cause that participants would have chosen to support regardless of whether empathy was induced or not. Thus, the lack of significance could be because participants have strong, established perceptions of stillbirth and what is considered appropriate when it comes to sharing memories. The influence of the empathy induction, therefore, may have made no difference to those established views. However, those in the high empathy condition were potentially moved to give more after feeling the effect of empathy, supporting the idea that a public education campaign regarding stillbirth and its effects on families may be warranted in order to create a socially supportive environment to enable families to grieve.

As time passed, participants’ perceptions of the appropriateness of sharing memories decreased significantly. As the social distance between affected parents and other people increased, participants considered sharing memories of the baby as becoming less appropriate. Thus, while sharing memories with immediate family is both acceptable and expected of grieving parents, this concession decreases as the relationship between people grows. Therefore, if parents’ attempts to share memories or discuss their stillborn are received in a non-positive manner due to the amount of time that has passed since the death, or if there is an implication that they should be “over it by now”, it can result in the feeling that they cannot share in the future. It could also leave parents feeling that their status as parent of a stillborn is something they need to keep quiet. According to research though, parents who have experienced a loss like this simply want their feelings validated and their experiences acknowledged (e.g., Üstündağ-Budak et al., 2015). However, coupled with the “taboo” of stillbirth, talking with non-family members can be challenging, considering that many people simply do not know what to say to someone who has experienced such loss.

The literature highlights the importance of both social support and memory sharing opportunities (e.g., Diamond et al., 2012; Crawley et al., 2013) and these results illustrate the need for an education campaign to raise awareness of stillbirth and the importance memory sharing is for parents. As discussed by Muller and Thompson (2003), sharing memories helps the bereaved come to terms with the death. It also has the potential to help others understand their experience. More opportunities for bereaved parents to be recognized would therefore be beneficial. For example, Australia officially declaring 15 October Pregnancy and Infant Loss Awareness day aims to provide support, awareness, and education regarding those who have experienced the loss of a child. Changes such as these will hopefully facilitate the creation of a more socially supportive environment for families of stillborn babies.

### Limitations and Future Research

The recruitment process in the current study was skewed. More women than men participated (14.5%), and the majority of participants were married, in a *de facto* relationship, or living with their partner (61%), all of which limits its generalizability. There was a fairly normal distribution in age and level of education, however, each participant had access to the internet. Future studies may want to examine potential differences in empathy provided to and/or needed by men and women in relation to stillbirth and other traumatic events. However, such gender differences may be much smaller than has been suggested (MacGeorge et al., 2004) to the extent where they may be irrelevant.

In line with Batson et al.’s (2002) research, helping was measured before perceptions in order to avoid pressure to help the group to maintain consistency with a just-expressed attitude. This leads to the possibility of the participant expressing perceptions to align with their helping behavior due to self-perception. However, empathy did not have an effect on the perception measures and previous research (Batson et al., 1997) demonstrated empathy had effected attitudes without a helping intervention.

While this study demonstrated that high empathy influenced people to allocate money to help stillbirth parents, even the induction of empathy did not change their perceptions toward stillbirth or memory sharing. It is recommended that similar research be conducted with a more representative group of participants while investigating potential mediating and/or moderating variables that could influence results. Further recommendations include developing and trialing an educational stillbirth campaign to measure its effects on participants’ perceptions. Establishing more positive perceptions toward stillbirth parents could facilitate a safer, more accepting social environment for families to openly grieve, remember, and share memories of their babies leading to more positive psychological outcomes. Future campaigns aimed at educating and raising awareness of stillbirth within the general community, similar to those seen with sudden infant death syndrome, may be one positive step forward in eradicating the taboo of stillbirth (see e.g., Mitchell et al., 2011).

Ultimately, the empathy manipulation was unsuccessful in its attempt to create high and low empathy groups. Perhaps participants in the two experimental groups attempted to repress any sense of anxiety or distress they felt as a result of watching the video. However, this should not necessarily obviate the current results given that the high and low groups did experience increased empathy compared to the control group.

## Conclusion

The results of the current study indicate that, in general, people are empathetic toward those who have experienced stillbirth, a highly emotive event. However, parents need to be able to share memories and thoughts relating to their child, given the cathartic nature of sharing experiences. As such, eliminating the taboo surrounding stillbirth, as well as educating people about how to talk about child loss is warranted. Informing people via resources such as the Stillbirth Foundation Australia^1^ has the potential to encourage discourse about stillbirth and bring the topic into the public arena. This, then, can only serve to support vulnerable parents – those coming to terms with the death of their baby.

## Author Contributions

All authors listed have made a substantial, direct and intellectual contribution to the work, and approved it for publication.

## Conflict of Interest Statement

The authors declare that the research was conducted in the absence of any commercial or financial relationships that could be construed as a potential conflict of interest.

## Footnotes

1 http://stillbirthfoundation.org.au/
